# Costs incurred by patients with tuberculosis co-infected with human immunodeficiency virus in Bhavnagar, western India: a sequential explanatory mixed-methods research

**DOI:** 10.1186/s12913-022-08647-2

**Published:** 2022-10-20

**Authors:** Mihir P. Rupani, Sheetal Vyas

**Affiliations:** 1 Department of Community Medicine, Government Medical College Bhavnagar (Maharaja Krishnakumarsinhji Bhavnagar University), Near ST bus stand, Jail Road, Bhavnagar, Gujarat 364001 India; 2 Present address: Clinical Epidemiology, Division of Health Sciences, ICMR - National Institute of Occupational Health (NIOH), Meghaninagar, Near Raksha Shakti University, Ahmedabad, Gujarat 380016 India; 3 Gujarat University, Ahmedabad, Gujarat India; 4 Department of Community Medicine, AMC-MET Medical College, Maninagar, Ahmedabad, Gujarat 380008 India

**Keywords:** Social protection, Negative financial coping, Collaborative framework, Bidirectional activities, National tuberculosis elimination program, Universal cash transfers, India

## Abstract

**Background:**

India reports the highest number of tuberculosis (TB) and second-highest number of the human immunodeficiency virus (HIV) globally. We hypothesize that HIV might increase the existing financial burden of care among patients with TB. We conducted this study to estimate the costs incurred by patients with TB co-infected with HIV and to explore the perspectives of patients as well as program functionaries for reducing the costs.

**Methods:**

We conducted a descriptive cross-sectional study among 234 co-infected TB-HIV patients notified in the Bhavnagar region of western India from 2017 to 2020 to estimate the costs incurred, followed by in-depth interviews among program functionaries and patients to explore the solutions for reducing the costs. Costs were estimated in Indian rupees (INR) and expressed as median (interquartile range IQR). The World Health Organization defines catastrophic costs as when the total costs incurred by patients exceed 20% of annual household income. The in-depth interviews were audio-recorded, transcribed, and analyzed as codes grouped into categories.

**Results:**

Among the 234 TB-HIV co-infected patients, 78% were male, 18% were sole earners in the family, and their median (IQR) monthly family income was INR 9000 (7500–11,000) [~US$ 132 (110–162)]. The total median (IQR) costs incurred for TB were INR 4613 (2541–7429) [~US$ 69 (37–109)], which increased to INR 7355 (4337–11,657) [~US$ 108 (64–171)] on adding the costs due to HIV. The catastrophic costs at a 20% cut-off of annual household income for TB were 4% (95% CI 2–8%), which increased to 12% (95% CI 8–16%) on adding the costs due to HIV. Strengthening health systems, cash benefits, reducing costs through timely referral, awareness generation, and improvements in caregiving were some of the solutions provided by program functionaries and the patients.

**Conclusion:**

We conclude that catastrophic costs due to TB-HIV co-infection were higher than that due to TB alone in our study setting. Bringing care closer to the patients would reduce their costs. Strengthening town-level healthcare facilities for diagnostics as well as treatment might shift the healthcare-seeking of patients from the private sector towards the government and thereby reduce the costs incurred.

**Supplementary Information:**

The online version contains supplementary material available at 10.1186/s12913-022-08647-2.

## Introduction

India is estimated to report the highest number of incident cases of tuberculosis (TB) globally [1]. In 2020, India notified 1.5 million cases of TB out of the total 5.8 million in the world [1]. The World Health Organization (WHO) called upon countries to eliminate TB by the year 2030 [2]. However, India is resolute to eliminate it by the year 2025 [3, 4]. In the year 2020, the number of people living with human immunodeficiency virus (HIV) was estimated to be 2.3 million in India - the second highest of the globally reported 37.7 million [5, 6]. An estimated 92,000 individuals (3.4%) were suffering from TB-HIV co-infection in India, accounting for 9% of the global burden in 2019 [7].

The national tuberculosis program in India has now been renamed as National Tuberculosis Elimination Program (NTEP) to eliminate TB by the year 2025. TB case finding and treatment activities are provided through a network of peripheral health institutions (PHIs), which includes health facilities run by the government, private, and non-governmental organizations. A TB unit (TU) has been set up at every taluk (town) for disease control activities at the sub-province level. Apart from a designated Medical Officer, the TB units are supported by supervisory staff, the senior treatment supervisors in rural areas, and TB health visitors in urban areas. After the initiation of treatment at a government-run PHI, care, and support in the form of monthly home visits is provided through the supervisory staff and peripheral health workers. The treatment is divided into the initiation and continuation phases, the total duration of treatment for drug-sensitive pulmonary regimen being of 6 months and that for drug-resistant pulmonary regimen ranging from 8 to 24 months. The duration of treatment for an extra-pulmonary regimen is guided by clinical improvement. Thus, the care for tuberculosis in India is largely decentralized, with the patients required to visit the PHIs only in case of any adverse drug reactions, complications, or follow-up sputum/other investigations.

The National AIDS Control Program (NACP) provides care, support, and treatment to patients with HIV in India through a network of Anti-Retroviral Therapy (ART) centers in the district places and link-ART centers at some taluks. The treatment, once started, continues lifelong with the patients required to visit the ART centers at least once a month for refilling their medicine stock and other laboratory investigations. The NACP does not have the ‘home-delivered care’ model that exists for NTEP, mainly due to the higher perceived stigma of HIV among the patients/community.

TB is the leading cause of death among people living with HIV, at the same time, HIV increases the susceptibility of patients to develop active TB disease [8]. To detect and manage patients with TB/HIV early, collaborative activities between NTEP and NACP have been implemented in India since the year 2012 [9]. The collaborative framework calls for each newly diagnosed TB patient to be referred for HIV testing and treatment, and vice versa. To reduce the number of clinic visits for patients with TB-HIV co-infection, medicines for both diseases are given through ART centers using a ‘single-window’ approach.

To supplement the dietary needs and serve as a financial cushion for patients with TB, the government of India launched a cash assistance scheme in April 2018 that provides them with Indian Rupees (INR) 500 [~US$ 7] per month [10]. Patients with TB who belong to castes with a higher probability of having lower incomes are entitled to an additional INR 500 [~US$ 7] per month for 6 months under a social welfare scheme in Gujarat. For patients with multi-drug resistant TB and patients with HIV, the transport fare of the patient and one accompanying member is reimbursed for the tickets of government-run means of transportation [11, 12].

One of the goals to be achieved by the year 2030 is to reduce the catastrophic costs incurred by patients due to TB to zero [2]. In India, the prevalence of catastrophic costs ranges from 4 to 68%, depending on the study setting, type of provider (government vs. private), and type of TB [13, 14]. Studies estimating patient costs due to TB-HIV have been reported from South Africa, Ethiopia, and Brazil [15–18]. Studies have reported higher patient costs as well as higher catastrophic costs for combined care of TB-HIV when compared with TB alone (64% vs. 47%) [15, 16]. However, one study also reported lower costs (as a percentage of annual household income) for patients with TB-HIV co-infection as compared with TB alone, but, reported higher mean total costs [17].

The bi-directional screening and management of patients with TB/HIV have been in place in India for over a decade now. Evidence estimating the costs among patients with TB-HIV co-infection is scarce in India. We anticipated that care for HIV would increase the financial burden on patients with TB. Our research has the potential to inform policy changes aimed at reducing TB’s catastrophic costs to zero among TB-HIV co-infected patients. We conducted this study to estimate the costs incurred by patients with TB co-infected with HIV in the Bhavnagar region of western India and to explore the perspectives of patients as well as program functionaries on reasons and solutions for reducing the costs.

## Methods

### Study design and setting

We conducted a sequential explanatory mixed-methods research among patients with TB co-infected with HIV in the Bhavnagar district of Gujarat state (western part of India). A descriptive cross-sectional study was followed by in-depth interviews. Bhavnagar district, with a population of ~ 2.8 million, is largely a rural area with urban conglomerates in certain provinces. Bhavnagar city (under the municipal corporation) has a population of ~ 0.6 million. The descriptive cross-sectional study was conducted to estimate the costs incurred by patients with TB co-infected with HIV. The purpose of the in-depth interviews was to explore the perspectives of the program functionaries and patients with TB co-infected with HIV on the reasons and solutions for the increased costs (constructivist paradigm). We used a descriptive design - a description of the codes and categories - for explaining the perspectives.

### Study population and duration

#### Quantitative

We included all adult patients with tuberculosis co-infected with HIV notified in the public sector between January 2017 and December 2020 in the Bhavnagar region of Gujarat state. We excluded patients under the age of 18, those who had been re-treated for TB, those with diabetes, those who had been notified in the private sector, and those who refused to participate in the study. The data collection was done between July 2019 and January 2021.

#### Qualitative

The in-depth interviews were continued till the saturation of responses was achieved. We included 17 program functionaries - 1 district TB officer, 1 district program coordinator, 9 senior treatment supervisors, and 6 TB health visitors; and 8 TB-HIV co-infected patients for the in-depth interviews. The interviews were conducted in February–May 2021.

### Data collection tool

#### Cost survey tool (quantitative)

The cost survey was conducted using a tool adapted from the World Health Organization’s validated questionnaire on estimating costs incurred by patients with TB [14, 19]. Socio-demographic information and questions on coping strategies employed by the patients were also added to the tool.

#### Interview guide (qualitative)

An interview guide focusing on the reasons and solutions for the increased costs incurred by patients with TB co-infected with HIV was prepared. Appropriate probing questions on delay in symptom identification, accessibility to health facilities, adverse drug reactions, staff involvement, and others were added to the interview guide.

### Data collection

#### Quantitative

The list of patients meeting the eligibility criteria was accessed from the Nikshay online platform (digital data entry of the treatment registers of patients with TB). The district TB officer of Bhavnagar provided the list of patients with TB notified during 2017–2020 in the form of an Excel sheet from the Nikshay portal. For preparing the final list of eligible study participants, we used relevant filters for age, HIV status, type of case (new vs. retreated), diabetes status, and patients notified in the private sector. The patients were visited at their homes to confirm eligibility, the informed consent procedure, and the cost survey. For patients who were inaccessible due to the COVID-19 lockdown, the data was collected by contacting them on the phone. During the home visit of patients, the TB health visitors and senior treatment supervisors were asked to accompany them for locating the homes and for building rapport. The patients were followed-up passively and their treatment outcomes were accessed from the data shared by the district officials from the Nikshay portal.

Based on patient recall, we collected data on costs for TB from the time of onset of symptoms of TB till treatment completion (including costs incurred for visits for adverse drug reactions, medicine collection visits, and other follow-up visits). The survey covered all costs up until the time of entry in the study for participants receiving TB treatment. We later conducted phone calls, depending on how far the patient was in the therapy phase, to collect data on the additional costs incurred till treatment completion. Costs for HIV were estimated for the diagnosis and treatment of HIV, and the follow-up medicine collection visits till the treatment completion of TB.

#### Qualitative

Both the authors are trained in qualitative research methods. The program functionaries and patients with TB-HIV who were perceived as being knowledgeable and vocal were purposively selected for the in-depth interviews. The in-depth interviews were conducted at a time convenient to them, after explaining to them the purpose of the interviews. All the interviews were audio-recorded in the local vernacular language (Gujarati).

### Definitions (quantitative)

#### Costs, catastrophic costs, and combined costs

Costs were categorized into direct medical costs (hospital stay charges, consultation charges, charges for radiography, laboratory and other procedures, and charges for drugs, and any prescribed nutrition), direct non-medical costs (travel fare, charges for food and accommodation, and opportunity cost - costs paid for household chores during hospital visits), and indirect costs (loss of wages and income loss - family income before TB minus after TB). The total costs are the sum of direct medical, direct non-medical, and indirect costs (subtracting money received through cash assistance or any reimbursement). Costs due to TB are defined as catastrophic when the total costs for TB exceed 20% of the annual household income [19]. We calculated combined TB-HIV costs by adding each category of costs for TB with the respective category of costs due to HIV. During the period of data collection, the average exchange rate of one US dollar (US$) was equal to 68 Indian rupees.

#### Standard of living index and annual household income

The standard of living index was calculated from the data of assets held by the patients such as type of house, number of rooms, car/truck, and others (Additional file 1) [14, 20, 21]. The scores ranged from 1 to 23, with a score of 1–7 grouped as low SLI and ≥ 8 as middle/high SLI [14]. We estimated annual household income by multiplying the self-reported monthly income of the family by 12.

#### Treatment outcomes

Unfavorable treatment outcomes are defined when a patient with TB tests positive by smear or culture at the end of treatment (treatment failure), interrupts treatment continuously for ≥1 month (lost to follow up), or, dies during the course of treatment (death) [22]. Successful treatment outcomes are defined when a patient with TB tests negative by smear or culture at the end of treatment (cured), or, has completed treatment without evidence of failure or clinical deterioration (treatment completed) [22].

### Analysis

#### Quantitative

The costs were summarized as median (interquartile range IQR) using the Statistical Package for Social Sciences (SPSS) software version 23 and expressed in Indian rupees (INR).

#### Qualitative

The audio-recorded interviews were transcribed into the English language in a Word document. Codes were assigned to the transcription and entered into an Excel sheet. Thematic analysis was used to generate categories from the codes. Since the codes generated were exhaustive, we used the ‘Framework Method’ to present our findings [23].

## Results

### Quantitative

#### Characteristics of patients

**Fig. 1:**
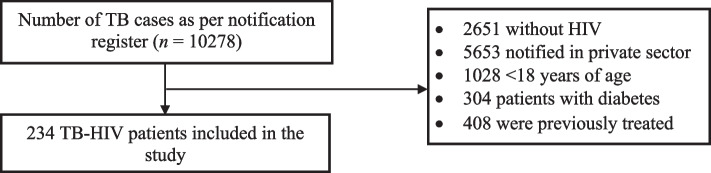
Selection process of patients notified with tuberculosis co-infected with HIV from January 2017 to December 2020 in Bhavnagar

We included 234 patients with TB co-infected with HIV in our study (100% response rate) (Fig. 1). Among the 234 study participants, 78% were male, 53% resided in an urban area, 19% had a low SLI index, and 18% were sole earners in their families (Table 1). Twenty percent of the study participants experienced unfavorable treatment outcomes, 39% had their first visit to a private provider for TB and 24% had it for HIV.

**Table 1:** Characteristics of patients with tuberculosis co-infected with HIV from January 2017 to December 2020 in Bhavnagar (*n* = 234)

Characteristic	Number (%) or median (IQR)
**Socio-demographic characteristics**	
Age in years	37 (29–48)
Male	182 (78)
Educational status	
No formal education	156 (68)
Primary (7th pass)	59 (25)
Secondary (10th pass) and above	19 (8)
Married	184 (79)
Scheduled caste (SC)/scheduled tribe (ST)	20 (9)
Extended family (vs nuclear family)	186 (80)
Urban residence	125 (53)
Current tobacco smoking	65 (28)
Current regular alcohol consumption	3 (1)
**Economic characteristics**	
Cash assistance received for TB (*n* = 139)	97 (70)
Amount of cash assistance received in INR (*n* = 139)	3000 (0–3000) [~US$ 44 (0–44)]
Monthly family income in INR	9000 (7500–11,000) [~US$ 132 (110–162)]
Below poverty line (BPL) card	117 (50)
Standard of living (SLI) index	
Low (SLI score 1–7)	44 (19)
Middle/high (SLI score 8–23)	190 (81)
Employed in paid work before TB diagnosis	144 (62)
Currently in paid work	122 (52)
Sole earner in the family	41 (18)
**Clinical characteristics - TB**	
Sputum acid-fast bacillus smear grade	
Negative	170 (73)
Scanty	22 (9)
1+	27 (12)
2+	10 (4)
3+	5 (2)
Extrapulmonary TB	66 (28)
Drug-resistant TB	9 (4)
Phases of treatment	
Intensive phase of TB treatment	25 (11)
Continuation phase of TB treatment	51 (22)
Treatment completed	158 (67)
First TB visit with a private provider	91 (39)
Hospitalized due to TB at the first visit	30 (13)
Treatment outcomes	
Successful treatment outcomes	
Treatment completed	43 (18)
Cured	143 (61)
Unfavorable treatment outcomes	
Death while on treatment	33 (14)
Lost to follow up	8 (3)
Treatment failure	7 (3)
**Clinical characteristics - HIV**	
On ART	224 (96)
CD4 count in mm^3^	350 (218–420)
First HIV visit with a private provider	55 (24)
Hospitalized due to HIV at the first visit	16 (7)

*IQR* Interquartile Range, *TB* Tuberculosis, *HIV* Human Immunodeficiency Virus

#### Costs incurred due to TB and TB-HIV

The total median (IQR) costs incurred for TB were INR 4613 (2541–7429) [~US$ 69 (37–109)], which increased to INR 7355 (4337–11,657) [~US$ 108 (64–171)] on adding the costs due to HIV (Table 2). The median (IQR) direct medical costs were higher for TB [0 (0–1043)] [~US$ 0 (0–15)] as compared to HIV [0 (0–0)]. While the medians are equal for both groups, the direct medical costs due to TB skewed higher than that due to HIV, with 75th percentile values of INR 1043 (US$ 15) and 0, respectively. The hospital stay charges, laboratory charges, and costs of medicines mainly contributed to the direct medical costs for TB. Travel costs to attend the health facilities and patient wage loss mainly contributed to the direct non-medical and indirect costs, respectively, for TB as well as for HIV. The median (IQR) direct non-medical costs for TB-HIV [3195 (2018–5168)] [~US$ 47 (30–76)] were higher than for TB [1970 (1355–3593)] [~US$ 29 (20–53)].

**Table 2:** Median (IQR) costs in Indian Rupees incurred by patients with TB-HIV co-infection from January 2017 to December 2020 in Bhavnagar (*n* = 234)

Categories of costs	TB costs	HIV costs	Combined TB-HIV costs
Direct medical	0 (0–1043) [~US$ 0 (0–15)]	0 (0–0)	0 (0–1700) [~US$ 0 (0–25)]
Hospital stay charges	0 (0–100) [~US$ 0 (0–1.5)]	0 (0–0)	0 (0–113) [~US$ 0 (0–1.7)]
Consultation	0 (0–0)	0 (0–0)	0 (0–0)
Radiography	0 (0–0)	0 (0–0)	0 (0–0)
Laboratory	0 (0–300) [~US$ 0 (0–4)]	0 (0–0)	0 (0–300) [~US$ 0 (0–4)]
Procedures	0 (0–0)	0 (0–0)	0 (0–0)
Drug	0 (0–500) [~US$ 0 (0–7)]	0 (0–0)	0 (0–1000) [~US$ 0 (0–15)]
Prescribed nutrition	0 (0–0)	0 (0–0)	0 (0–0)
Direct non-medical	1970 (1355–3593) [~US$ 29 (20–53)]	1080 (660–1500) [~US$ 16 (10–22)]	3195 (2018–5168) [~US$ 47 (30–76)]
Travel to attend health facility	1970 (1355–3550) [~US$ 29 (20–52)]	1055 (660–1500) [~US$ 16 (10–22)]	3175 (2018–5163) [~US$ 47 (30–76)]
Food purchased to attend health facility	0 (0–0)	0 (0–0)	0 (0–0)
Accommodation to attend health facility	0 (0–0)	0 (0–0)	0 (0–0)
Opportunity costs during clinic visits	0 (0–0)	0 (0–0)	0 (0–0)
Indirect	1252 (683–2192) [~US$ 18 (10–32)]	725 (338–1600) [~US$ 11 (5–24)]	2218 (1234–3690) [~US$ 33 (18–54)]
Loss of wages to attend health facility	824 (331–1300) [~US$ 12 (5–19)]	400 (138–1200) [~US$ 6 (2–18)]	1386 (650–2351) [~US$ 20 (10–35)]
Wage loss of accompanying member	300 (150–500) [~US$ 4 (2–7)]	200 (200–400) [~US$ 3 (3–6)]	600 (300–900) [~US$ 9 (4–13)]
Household income loss due to TB	0 (0–0)	–	–
Total costs	4613 (2541–7429) [~US$ 69 (37–109)]	2223 (1278–3825) [~US$ 33 (19–56)]	7355 (4337–11,657) [~US$ 108 (64–171)]

*IQR* Interquartile Range, *TB* Tuberculosis, *HIV* Human Immunodeficiency Virus

#### Catastrophic costs and coping strategies

The catastrophic costs at a 20% cut-off of annual household income for TB were 4% (95% CI 2–8%), which increased to 12% (95% CI 8–16%) on adding the costs due to HIV (Additional file 2: Supplementary Table 1. Despite the lower percentages of catastrophic costs incurred by the patients, 15% had to employ a coping strategy to overcome the increased costs (Table 3). Nine percent of the patients lost employment after diagnosis of TB, 2% borrowed money as a loan and the median (IQR) days of work lost to TB was 45 (29–70).

**Table 3:** Coping strategies employed by patients with TB co-infected with HIV from January 2017 to December 2020 in Bhavnagar (*n* = 234)

Type of coping strategy	Number (%) or median (IQR)
Coping strategy of any one kind	34 (15)
Borrowed money as loan	5 (2)
Amount borrowed in Indian Rupees	10,000 (1501–15,000)
Lost employment after TB diagnosis	21 (9)
Started employment to cover costs of TB	3 (1)
Working days lost to TB	45 (29–70)
Outsourced household chores during TB care	5 (2)

*IQR* Interquartile Range, *TB* Tuberculosis, *HIV* Human Immunodeficiency Virus

### Qualitative

**Fig. 2:**
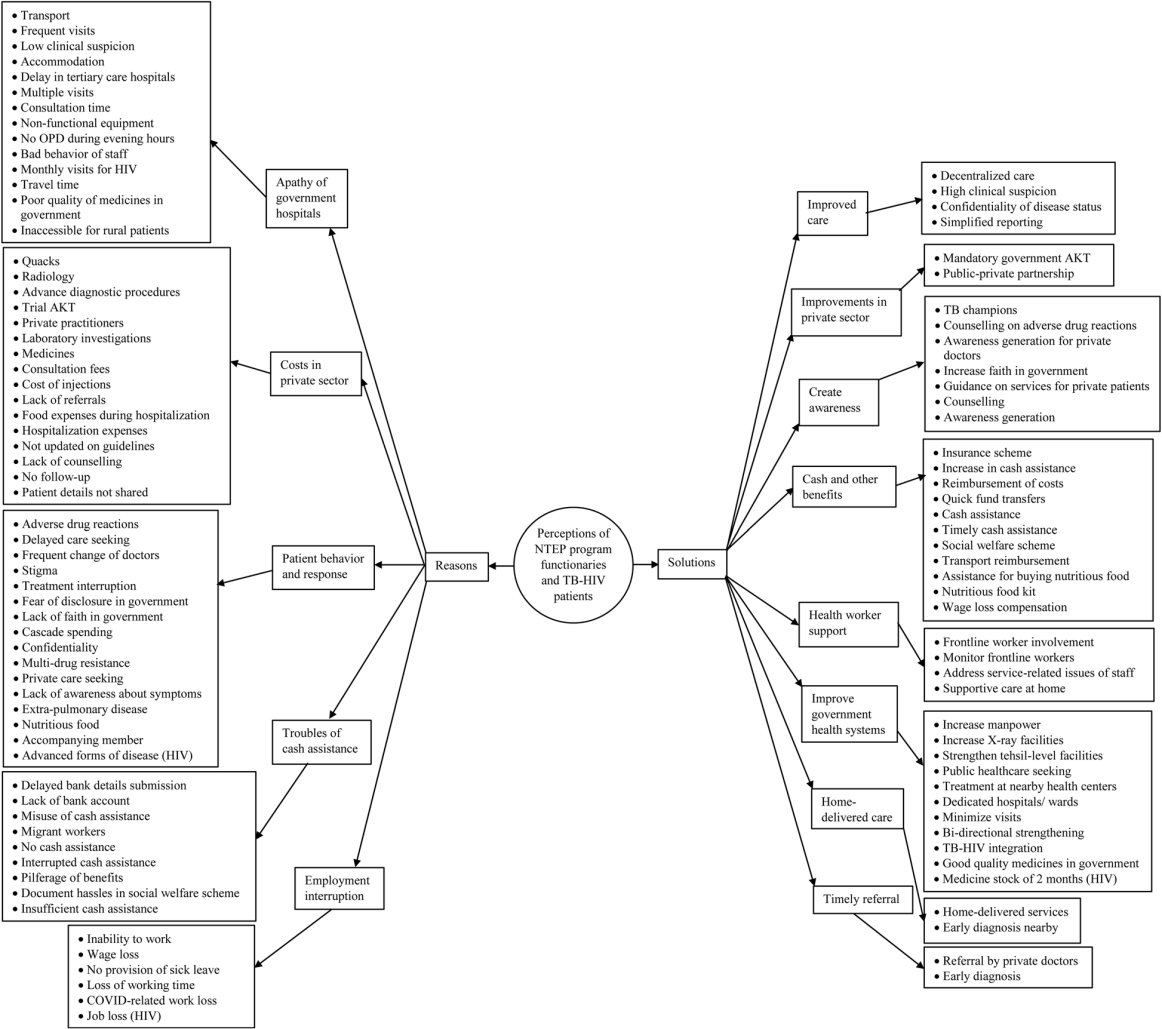
**a** Reasons for increased costs due to TB and HIV as perceived by program functionaries and patients in Bhavnagar. **b** Solutions for increased costs due to TB and HIV as perceived by program functionaries and patients in Bhavnagar

The median (IQR) experience of the 17 program functionaries was 7 (5–20) years; 3 were female. Among the eight patients with TB-HIV, the median (IQR) age was 42 (26–49) years; one was a female. The codes generated from the analysis of the transcript of in-depth interviews were arranged as a code tree (Fig. 2a and b).

#### Reasons for increased costs of TB-HIV

The perceptions of the program functionaries as well as the patients were comparable for the reasons of increased costs due to TB-HIV (Table 4). The reasons for increased costs were described under the categories - apathy of government hospitals, costs in the private sector, patient behavior and response, troubles with cash assistance, and employment interruption (refer to Additional file 3: Supplementary Table 2 for a detailed explanation of each code).

**Table 4:** Comparison of categories generated for reasons of increased costs of TB-HIV from perceptions of program functionaries and patients using the framework method

Categories	Program functionaries		Patients	
	TB	HIV	TB	HIV
Apathy of government hospitals	√	√	√	√
Costs in private sector	√	√	√	√
Patient behavior and response	√	√	√	√
Troubles with cash assistance	√	√	√	√
Employment interruption	√	√	√	√

##### Apathy of government hospitals

Despite the free treatment at government hospitals, patients incur costs for transport, frequent visits, and delays in care. For the care of HIV, the major reason was the lack of decentralized care and the inaccessibility of tertiary care hospitals for patients residing in rural areas.

*“For diagnosis, the patients have to do frequent visits, immediately reports are not available, plus they have to waste a lot of time. Apart from the problems faced by them for transport, if they get some comfort after coming here that proper diagnosis was done, a proper examination was done, proper treatment was given … because sometimes patients do complain that doctor was not available, or proper diagnosis was not done or timely report was not available or for one report they had to wait for 4-5 hours from morning till evening - these kind of complaints are there.”* (TB health visitor, 5 years of experience).

*“For ART, even patients in rural areas have to come to Bhavnagar* [tertiary care hospital] *for getting their monthly medicines.”* (Senior treatment supervisor, 18 years of experience).

##### Costs in private sector

Costs incurred due to consultation, medicines, investigations, and others due to visits to private practitioners were pertinently adding to their total costs. Private practitioners, despite being aware of free treatment in government, did not counsel/refer patients or, used to give anti-TB therapy on a trial basis, ultimately leading to increased costs.

*“I used to remain sick so we used to take medicines from a doctor nearby. I had diarrhea and vomiting, so the doctor couldn’t recognize* [my disease]*. Then I went to a surgeon at a hospital, who charged me 7000 rupees for 2 days. We spent 1200-1500 rupees on consultation. After doing the reports in another hospital, they referred me to a government hospital as the expenses would have been 10000-15000 rupees per month.”* (59 years, male patient).

*“There are 25% patients whose counseling is not done or, sometimes even there are 10% patients who feel that I took treatment for 10-15 days, and I am feeling better now. I am spending my money, losing my daily wages by putting leave, I am spending 1500-1700 rupees, so I do not need any further medicines.”* (Senior treatment supervisor, 5 years of experience).

##### Patient behavior and response

Adverse drug reactions, treatment interruption, multi-drug resistance, stigma, fear of disclosure, lack of awareness, and private care-seeking were some of the patient-related reasons for the increased costs among the co-infected patients.

*“Patients make special requests that they don’t want to declare. Not disclosing is the stigma toward TB. People do not go to the government, they go to private. They believe that TB is inherited, so it will be transmitted to their children. That is why they go to private.”* (Senior treatment supervisor, 20 years of experience).

*“We went to the government hospital first and there was some recovery. In the middle of the treatment, I stopped the medicine as there were so many ulcers in my mouth. I got so sick that we went to the private hospital then.”* (42 years, female patient).

##### Troubles with cash assistance

Lack of bank account, document hassles, and interrupted/insufficient/no cash assistance were some of the reasons perceived for the troubles in receiving the monthly cash assistance among patients with TB. Although there is no cash assistance scheme for patients with HIV, except reimbursement of transport fare, it was perceived that co-infected patients should receive higher cash assistance under the TB program.

*“I was told that 500 rupees per month will be given but, it was given only for one month and not after that. My livelihood can continue if we get proper assistance. I am the sole earning person in my family, my son is young and he studies and does menial jobs after school. My wife too does cooking-related work.”* (49 years, female patient).

“*… if he is very poor or is a migrant worker, he does not have an Aadhaar card* [social security number]*, then it happens... Or, if a patient who even didn’t open a bank account, then they do not get the cash assistance.”* (Senior treatment supervisor, 3 years of experience).

##### Employment interruption

Inability to work during the debilitating TB-HIV co-infection was perceived as the primary reason for unemployment and subsequent loss of wages. The wage loss was contributed by the lack of provision of sick leaves during the hospital visits by the patients.

*“I lost my job due to HIV around 5 years ago. At our job they don’t grant us leave that causes a problem. Now I am searching for another job.”* (25 years, male patient).

*“Due to less immunity, due to weakness they are not able to work, since they are not able to work, the entire family’s income stops.”* (Senior treatment supervisor, 8 years of experience).

*“At the time of treatment, I took rest for 3 months continuous and could not go to work. I worked in a private company as a diamond worker so they did not pay my salary.”* (49 years, male patient).

#### Solutions for increased costs of TB-HIV

The solutions provided by the program functionaries were more aligned toward health system strengthening, whereas availing benefits, and reducing costs through timely referral were the solutions provided by the patients (Table 5). Cash and other benefits, and improvement in government health systems were the common solutions provided by program functionaries as well as the patients (refer to Additional file 3: Supplementary Table 2 for a detailed description of each code).

**Table 5:** Comparison of categories generated for solutions to increased costs of TB-HIV from perceptions of program functionaries and patients using the framework method

Categories	Program functionaries		Patients	
	TB	HIV	TB	HIV
Improved care	√			
Improvements in private sector	√			
Create awareness	√	√		
Cash and other benefits	√	√	√	√
Health worker support	√			
Improve government health systems	√	√	√	√
Home-delivered care		√		
Timely referral			√	√

##### Improved care

Providing decentralized care through the network of PHIs, maintaining a high clinical suspicion, and confidentiality of disease status were suggested for reducing the costs.

*“For HIV, I suppose that if their ART is kept in the nearby health centers, then it can reduce their costs of traveling every month to the main hospital.”* (TB health visitor, 10 years of experience).

*“We have to convince them that we will not come to their house, but take government medicine, no one should know around us. With that commitment, they take our medicine. We don’t tell anyone, we don’t visit their house, this way we have completed many patient’s treatments.”* (Senior treatment supervisor, 8 years of experience).

##### Improvements in private sector

Mandatory government-provided drugs, and fostering public-private partnerships were the solutions suggested concerning the private sector.

*“Government should make sure that any doctor, including private, has to give AKT or MDR medicine from the government only. The patient should not be able to change the medicine on his own. There is a DST-guided* [drug-susceptibility testing] *treatment in government.”* (Senior treatment supervisor, 20 years of experience).

*“If the government wants, they can give projects to NGOs* [non-governmental organizations]*. There were NGO hospitals, here also we had one but it is closed now. They used to test our sputum for free, HIV testing for free.”* (Senior treatment supervisor, 7 years of experience).

##### Create awareness

Generating awareness for patients as well as for private practitioners on several aspects was suggested. Identifying ‘TB champions’, counseling on adverse drug reactions, increasing faith in government, and guidance on various services which can be availed by patients being treated in the private sector were suggested for creating awareness and subsequently reducing costs among the patients.

*“We form a TB champion group comprising of village leaders, health workers, and volunteers. We give all the information related to TB to this group. If any patient in the village is refusing TB treatment, then the champion group takes the help of a patient who has completed treatment and counsels non-compliant patients to complete their treatment. A cured patient can say that look at me I am cured now … they may believe him.”* (Senior treatment supervisor, 5 years of experience).

*“At present, the private and the government are in a mix, so we can give enough guidance. We can explain to a private patient on a phone call. We can guide HIV, and diabetes patients also. After taking the Aadhaar card* [social security number] *and bank details, we explain to them about 500 rupees assistance per month even in private.”* (TB health visitor, 7 years of experience).

##### Cash and other benefits

Reimbursement of costs/losses, increasing cash assistance, provision of nutritious food kits and quick fund transfers were the solutions suggested for reducing the costs. Linking with existing social security schemes such as Prime Minister’s Jan Arogya Yojana (PM-JAY), a health coverage scheme for the vulnerable, was also suggested.

*“I believe they incur costs for food. We ask them to eat healthy food. People who spend their day just eating milk and bread … even for basic food, 500 rupees is not enough. Either we should provide them with a food kit or more money should be given in some way.”* (District program coordinator, 1.5 years of experience).

*“There should be an integration of NTEP with PM-JAY. If patients who are taking treatment from the private sector are included under PM-JAY, then they will incur fewer costs, they can also keep their disease status confidential.”* (Senior treatment supervisor, 20 years of experience).

##### Health worker support

Involving the frontline workers of the PHIs in providing supportive care at home, and establishing a mechanism of monitoring their work would ensure to reduce the burden on the existing health visitors under the TB program. Most of the frontline workers are posted at the village level or, nearby human settlements in urban areas, making them potentially well-placed to act as supporters of programs for TB and HIV.

*“In rural areas, the PHI staff should be given the responsibility to provide care at home. For that, our supervisory team should be given the power to monitor. So that I can take some action if they don’t do the work.”* (Senior treatment supervisor, 20 years of experience).

*“If HIV is suspected, then ASHA worker* [village-level health worker] *can inform a village leader, and then they can inform us accordingly. If there are symptoms of TB or HIV, then it is common to get sick again and again, so they can inform us. If any person is not cured by the treatment as stated, and if they inform us, then we will send their samples for further investigations.”* (Senior treatment supervisor, 4 years of experience).

##### Improve government health systems

Strengthening tehsil-level (town-level) health facilities with the provision of digital X-ray facilities and additional manpower, minimizing visits through the strengthening of the bi-directional TB-HIV collaborative activities, and through the provision of HIV drug stock of 2 months (instead of monthly stock) were some of the suggestions for improving the government health system for reducing the costs incurred by co-infected patients.

*“Currently, there is only one technician in a primary health center. If there are two technicians, one specially dedicated for TB, then an early diagnosis can be done.”* (Senior treatment supervisor, 20 years of experience).

*“In government, they are not having digital X-rays, they have very old machines. They are providing poor X-rays, so our MO* [Medical Officer] *is not able to identify early whether these are Koch’s lesions or something else. If the government gives digital X-ray facilities, then patients will be benefitted a lot.”* (Senior treatment supervisor, 7 years of experience).

*“If they can provide the medicine* [for HIV] *for 2-3 months, then we need not visit the hospital monthly … our expenses would be less and the patient load would also decrease. If there is any problem, we would go for a check-up.”* (59 years, male patient).

##### Home-delivered care

The program functionaries suggested that the diagnosis, treatment, and other required support for the care of HIV patients should be available nearby their homes for reducing the costs due to the requirement of monthly visits to a tertiary care hospital.

*“During Village Health & Nutrition Days* [monthly services provided by health workers in villages and urban settlements] *or any other such events, let the patient/anyone who has a problem be informed about the date of such events. There they can be diagnosed with HIV and other diseases, and reports can be sent to them. If such a notice is put on the notice board by the village leaders, then the public will be benefitted.”* (Senior treatment supervisor, 4 years of experience).

##### Timely referral

The patients perceived that early diagnosis and timely referral of non-affording patients to government health facilities by private practitioners would reduce their costs significantly.

*“If private doctors do not order too many reports and diagnose early, then we can save money. My doctor ordered too many reports and took around 2500 rupees, only then was he able to diagnose my disease. Early reports would be helpful.”* (26 years, male patient).

*“When I got sick, 3-4 years back then I got admitted in a private hospital and they charged around 5000-6000 rupees. Again, I got sick and went to another doctor, he charged 1500 rupees. I did not know about this problem* [HIV]*. But, he referred me to a government hospital for medicines.”* (59 years, male patient).

## Discussion

In our study setting, costs incurred due to combined care of TB-HIV were significantly higher than those for TB alone. Patients incurred lower catastrophic costs due to TB, which increased substantially on adding the costs incurred due to HIV. Even with lower catastrophic costs, nearly one in six patients had to employ a coping strategy, such as starting new employment to cover the costs of care. The need for improving facilities such as digital X-ray machines and increasing human resources, especially laboratory technicians, was suggested as one of the solutions for reducing the costs of TB. Providing care to patients with HIV nearer to their homes and reducing clinic visits by replenishing stock of 2–3 months’ medicines during each visit was also suggested.

The median (IQR) cost of TB in our study was INR 4613 (2541–7429) [~US$ 69 (37–109)]. This was less than the INR 10000 [~US$ 147] median cost revealed in a recent nationally representative survey in India [24]. We also found that 4% of patients faced catastrophic costs due to TB in our study setting. This finding was comparable to a few studies in India [14, 25], but lower than a majority of other studies conducted in metro cities, which reported a prevalence of up to 68% [13, 26]. Our study setting is primarily semi-urban and rural, with decentralized TB care, support, and treatment. There are very few villages that are > 3 miles away from a government health facility in Bhavnagar district, while in Bhavnagar city, the government health facilities are located within a mile’s distance. Except for initial diagnostic and follow-up visits, patients with TB in our study setting sought care from a government health facility nearest to them. Also, the drugs for TB were delivered to their homes through treatment supporters on monthly basis. In case of adverse drug reactions or any complications during the course of treatment, patients had an option to visit a government health facility near their homes. The decentralized model of care might explain the lower percentage of catastrophic costs due to TB in our study setting. However, two-fifths of the patients had their first clinic visit with a private practitioner. Also, for quick relief from the side effects of drugs, patients might have sought care from private practitioners. The care-seeking behavior of patients towards the private sector might explain the higher median costs incurred for TB as compared to HIV in our study setting.

We found in our study that 12% of patients faced catastrophic costs due to TB-HIV. There was no similar research from India on combined TB-HIV costs to support our study findings. However, investigators outside India reported a higher prevalence of catastrophic costs due to combined TB-HIV co-infection [15, 17], probably due to differing methods of defining catastrophic costs. In our study setting, the median (IQR) cost of combined TB-HIV care was INR 7355 (4337–11,657) [~US$ 108 (64–171)]. Patients, in our study setting, had to visit tertiary-level clinics when it came to caring for HIV. The drugs as well as certain specific investigations for HIV could only be availed at the tertiary care hospitals located in urban areas. Patients from rural areas had to travel long distances and also had to lose wages for the days of clinic visits for the care of HIV. Due to the stigma surrounding HIV disease, there were barriers to providing home-delivered care through healthcare workers. To maintain confidentiality, some patients might have sought care for HIV from private practitioners too. These reasons might have increased the cost of care for HIV in our study setting. This also explains the increase in the percentage of catastrophic costs for combined TB-HIV over TB alone.

The findings from the in-depth interviews pointed toward the need for strengthening town-level health care facilities to reduce the costs incurred by the patients for the care of TB and HIV. Making available facilities for chest X-ray, improving access to care, increasing awareness of TB, and improving treatment adherence has been shown to reduce costs as well as incidence/mortality due to TB [27, 28]. Due to the costs incurred for transportation and wage loss for monthly clinic visits for HIV, the patients in our study suggested a longer duration for replenishment of drugs. Providing services nearer to the patients has been shown to reduce patient costs [29]. The program functionaries also suggested strengthening the bi-directional TB-HIV collaborative activities, proven to be a cost-effective intervention in regions with a high prevalence of co-infection [30]. Although a few cash assistance schemes have been in place for TB and HIV in India, it has to be seen whether universal cash transfers would prevent patients from being pushed below the poverty line [31, 32].

This is the first study estimating costs among patients with TB-HIV co-infection in India. Patients might have faced difficulty in recalling the costs incurred by them, thus an underestimation of costs cannot be ruled out. We did not estimate the costs from the societal perspective, that is, costs borne by the hospitals/providers for this study due to the current focus on the reduction of catastrophic patient costs. The exclusion of patients with TB notified in the private sector from the study may have resulted in an underestimate of the costs incurred. Using self-reported rather than consumption or expenditure-based incomes to estimate household incomes in our study may have resulted in an underestimation of the percentage of catastrophic costs. Finally, we estimated the costs of HIV only for the duration of TB treatment. However, we believe the results would be helpful for other low- and middle-income countries with a high prevalence of TB and HIV.

## Conclusions

We conclude that catastrophic costs due to TB-HIV co-infection were higher than that due to TB alone in our study setting. Bringing care closer to the patients would reduce their direct non-medical as well as indirect costs. Strengthening town-level healthcare facilities for diagnostics as well as treatment might shift the healthcare-seeking of patients from the private sector towards the government. Further research is needed on the feasibility and cost-effectiveness of strengthening health systems and universal cash transfers for reducing catastrophic costs among patients with TB-HIV co-infection in India.

## Supplementary Information

**Additional file 1.**

**Additional file 2.**

**Additional file 3.**

## Data Availability

The datasets generated and/or analyzed during the current study are available upon a reasonable request to the corresponding author.

## Abbreviations

AKT: Anti-Koch’s Treatment
ART: Anti-Retroviral Therapy
CI: Confidence Intervals
COVID-19: Coronavirus Disease-2019
HIV: Human Immunodeficiency Virus
INR: Indian Rupees
IQR: Interquartile Range
MDR: Multi-drug Resistant
NACP: National AIDS Control Program
NTEP: National Tuberculosis Elimination Program
PHI: Public Health Institution
SLI: Standard of Living Index
TB: Tuberculosis
WHO: World Health Organization
