# Supplementary material for: Costs incurred by patients with tuberculosis co-infected with human immunodeficiency virus in Bhavnagar, western India: a sequential explanatory mixed-methods research
Source: BMC Health Serv Res. 2022 Oct 20;22:1268. doi: 10.1186/s12913-022-08647-2 (PMC9581761; doi:10.1186/s12913-022-08647-2)
Supplement: Supplementary file 1 — Additional file 1. [file 12913_2022_8647_MOESM1_ESM.doc]

**Calculation of the Standard of Living (SLI) index:**

The SLI index was calculated based on the ownership of the assets by the households. We used the following scoring for the SLI index in our study.

1. *Type of house and number of rooms:* a score of 4 for a house which has walls and roof made of cement concrete and has ≥2 rooms; a score of 3 for a house which has walls made of cement concrete but the roof is made of material other than cement concrete and having ≥2 rooms; a score of 2 for a house which has walls made of cement concrete but the roof is made of material other than cement concrete and having 1 room; a score of 1 for a house which has walls as well as the roof made of material other than cement concrete, irrespective of the number of rooms [a pucca house with ≥2 rooms gets a score of 4, a pucca house with <2 rooms get a score of 3, a semi-pucca house with ≥2 room gets a score of 3, a semi-pucca house with 1 room gets a score of 2, a kutcha house gets a score of 1].
2. *Separate kitchen:* a score of 3 for a house with a separate kitchen, otherwise 0.
3. *Liquified Petroleum Gas (LPG) as cooking fuel:* score of 1 for a household using, otherwise 0.
4. *Land usable for agriculture:* score of 4 for owning such a land, otherwise 0.
5. *Television, bicycle, mobile:* score of 1 each for owning them, otherwise 0.
6. *Refrigerator, motorcycle/scooter:* score of 2 each for owning them, otherwise 0.
7. *Car/truck:* score of 4 for owning one, otherwise 0.
