# Supplementary material for: Costs incurred by patients with tuberculosis co-infected with human immunodeficiency virus in Bhavnagar, western India: a sequential explanatory mixed-methods research
Source: BMC Health Serv Res. 2022 Oct 20;22:1268. doi: 10.1186/s12913-022-08647-2 (PMC9581761; doi:10.1186/s12913-022-08647-2)
Supplement: Supplementary file 2 — Additional file 2. [file 12913_2022_8647_MOESM2_ESM.doc]

Supplementary Table 1: Catastrophic costs incurred due to TB and TB-HIV at different cut-off percentage of annual household income from January 2017 to December 2020 in Bhavnagar (n=234)

| **Cut-off percentage of annual household income** | **Percentage (95% *CI*) of households facing catastrophic costs due to TB** | **Percentage (95% *CI*) of households facing catastrophic costs due to TB-HIV** |
| --- | --- | --- |
| 20 | 4 (2-8) | 12 (8-16) |
| 15 | 11 (7-15) | 17 (12-22) |
| 10 | 16 (12-22) | 31 (26-37) |
| 5 | 41 (35-47) | 66 (60-72) |

CI: Confidence Interval; TB: Tuberculosis; HIV: Human Immunodeficiency Virus
