# Supplementary material for: Costs incurred by patients with tuberculosis co-infected with human immunodeficiency virus in Bhavnagar, western India: a sequential explanatory mixed-methods research
Source: BMC Health Serv Res. 2022 Oct 20;22:1268. doi: 10.1186/s12913-022-08647-2 (PMC9581761; doi:10.1186/s12913-022-08647-2)
Supplement: Supplementary file 3 — Additional file 3. [file 12913_2022_8647_MOESM3_ESM.doc]

Supplementary Table 2: Description of reasons of increased costs incurred by patients with TB co-infected with HIV, as perceived by program functionaries and TB-HIV patients

| **Reasons for increased costs of TB-HIV** | | |
| --- | --- | --- |
| **Categories** | **Code** | **Description of code** |
| Apathy of government hospitals | Transport | Patients pay for transport fare for their clinic visits, higher costs incurred by rural patients |
|  | Frequent visits | Frequent visits for follow-up sputum tests, delay in lab reports, in case of adverse drug reactions, or any other complications |
|  | Low clinical suspicion | Medical Officers of public health institutions (PHIs) generally have low clinical suspicion for tuberculosis, thereby delaying case detection |
|  | Accommodation | Patients pay for accommodation during clinic visits, sometimes for their accompanying family member too |
|  | Delay in tertiary care hospitals | Tertiary care hospitals, being multi-disciplinary and multi-specialty, patients are unaware about the out-patient department or laboratory to go for consultation/ investigations. Patients have to spend their entire day at tertiary care hospitals, leading to loss of wages, which ultimately shifts their care-seeking behavior towards private sector |
|  | Multiple visits | If patients reach to government hospitals a bit late, they have to pay multiple visits as their reports get delayed or are made available on the next day |
|  | Consultation time | Patients have to wait their turn, especially when there is rush, leading to loss of their time at workplace |
|  | Non-functional equipment | If X-ray machines are not working on the day of visit, patients have to visit some other day |
|  | No OPD during evening hours | Lack of OPD during evening hours forced patients to lose their morning working time to visit hospitals or, seek care at private practitioners during evening hours |
|  | Bad behavior of staff | Patients have to face the bad behavior of staff at government hospitals. Patients do not talk back as they fear that the staff might give them wrong medicines if they show some restraint. |
|  | Monthly visits for HIV | Monthly medicine stock replenishment visits, generally to tertiary care hospitals, by patients with HIV |
|  | Travel time | Patients, especially those residing in rural areas, have to lose their time (generally it coincides with their time of going for work) in travelling to the government hospitals |
|  | Poor quality of medicines in government | Patients complain of poor quality (efficacy) of medicines in government hospitals, leading them to seek care in private sector |
|  | Inaccessible for rural patients | For rural patients, tertiary-care government hospitals become inaccessible due to the distance they have to travel. This increases their care-seeking towards the private sector. |
| Costs in private sector | Quacks | Patients in rural areas, sometimes prefer to consult quacks (unqualified doctors) as the first point of contact |
|  | Radiology | Private doctors generally rely on X-rays for their diagnosis of tuberculosis |
|  | Advance diagnostic procedures | For certain diseases like tuberculosis of organs other than lungs, advance diagnostic procedures such as fine-needle aspiration cytology are required |
|  | Trial AKT | Private practitioners start their treatment with a course of antibiotics, sometimes with trial AKT. The trial course provides early symptomatic relief to the patients, giving a false sense of cure. |
|  | Private practitioners | Private doctors continue to treat till the patients lose their ability to pay any more |
|  | Laboratory investigations | Costs for laboratory investigations in private sector |
|  | Medicines | Costs for drugs purchased from private doctors or chemists |
|  | Consultation fees | Fees paid to consult private doctors |
|  | Cost of injections | Multi-drug resistant patients have to pay for injectable drugs. This cost increases for HIV patients as the nurse prefers wearing gloves while giving injections. |
|  | Lack of referrals | Some private doctors are unaware that medicines for HIV are available for free in government due to which they do not refer patients |
|  | Food expenses during hospitalization | Costs incurred for food, mainly during hospitalization |
|  | Hospitalization expenses | Hospitalization bed expenses (day charges) |
|  | Not updated on guidelines | Guidelines change frequently for TB program, however private practitioners are not updated on the same. Yet, private care-seeking is observed among patients. |
|  | Lack of counselling | Paucity of time makes private practitioners to skip explaining about tuberculosis, type of TB, it’s duration of treatment, and others. |
|  | No follow-up | Private practitioners generally prescribe medicines for 15 days and ask patients to come for follow-up. However, if the patients do not turn up after 15 days, the private practitioners do not follow them up. This might lead to severe disease later on. |
|  | Patient details not shared | Private practitioners are not sharing details of the patients with the government If the private practitioners share patient details, then the government can provide the benefits of cash transfer scheme, and other benefits. |
| Patient behavior and response | Adverse drug reactions | Due to adverse drug reactions, patients pay an extra clinic visit, sometimes to private practitioners. |
|  | Delayed care seeking | Patients seek care only after the symptoms aggravate. Especially in rural areas, patients delay care seeking which leads to progression of disease at the time of diagnosis. |
|  | Change of doctors | If patients are not immediately relieved of their symptoms, they seek care from private doctors. They incur costs at private clinics, and when they are no longer able to spend money, they come back to government health centers. |
|  | Stigma | Patients, especially unmarried and female, are worried about their identity getting disclosed in community and thereby visit private doctors for care. The primary reason for the stigma is the potential harm to prospects of marriage and employment. This stigma is higher among patients with HIV, as compared with TB. |
|  | Treatment interruption | Patients who get free of symptoms in 10-15 days perceive that they are cured and tend to stop treatment. Some patients stop treatment due to adverse drug reactions and lack of information about completing treatment. Some tend to seek care at local healers or private practitioners in search of an early cure |
|  | Fear of disclosure in government | Patients fear that their disease status would get disclosed if they visit government hospitals, thereby they tend to seek care in private sector |
|  | Lack of faith in government | Irrespective of improvements in the quality of care in government facilities, there is resistance among the patients to seek care in government due to a general lack of faith |
|  | Cascade spending | Patients first seek care from a local unqualified doctor nearby. When not relieved of symptoms, they go to a tehsil-level private facility where they incur costs for investigations and X-ray. They take treatment for 10-15 days and soon run out of money. They take loan or borrow money and continue treatment at the private facility. Finally, they inform the private doctor of their inability to pay any further. This is the time when the private doctors inform them that these medicines can be availed for free in government health centers. |
|  | Confidentiality | Patients seek care at private health clinics to maintain confidentiality. |
|  | Multi-drug resistance | Multi-drug resistant TB regimen, being of longer duration and severe, patients tend to incur higher costs. |
|  | Private care seeking | It is a general mentality among the patients to first seek care from private doctors, and only when they are not satisfied with the results, they seek care from government health facilities. |
|  | Lack of awareness about symptoms | The lack of awareness about symptoms of TB/ HIV delays their care seeking. They generally seek care only when the symptoms aggravate or when disease progresses. |
|  | Extra-pulmonary disease | The diagnosis and treatment for extra-pulmonary disease is complex, thereby requiring more number of visits to health facilities and higher costs for its care. |
|  | Nutritious food | To maintain immunity and tolerate the adverse drug reactions, patients spend money on consumption of nutritious food during the period of treatment. |
|  | Accompanying member | Costs are incurred for the accompanying family member or relative during hospital visits/ stay. |
|  | Advanced forms of disease (HIV) | Progression of HIV tend to increase their visits to the clinics. |
| Troubles of cash assistance | Delayed bank details submission | Delay in submission of bank account number and Aadhaar card (social security number) details delays the processing of cash assistance under TB program |
|  | Lack of bank account | Patients not having a bank account are not able to avail of the cash benefit as it is a direct benefit transfer scheme |
|  | Misuse of cash assistance | Patients use the cash assistance in purchase of addictive substances |
|  | Migrant workers | Migrant workers do not have an Aadhaar card (social security number) due to their constant migration from one place to another, thereby making them ineligible for the cash benefit |
|  | No cash assistance | For cash assistance for HIV, a patient was asked to open a bank account with a credit of 3000 Indian rupees to receive the cash assistance. Also, patients complain of cash assistance not being received in their bank accounts under the TB program. |
|  | Interrupted cash assistance | Cash assistance for the TB program was perceived as being interrupted and not as per the schedule as defined under the program |
|  | Pilferage of benefits | Patients perceived that many benefits are being provided by the government, but not all reaches to them |
|  | Document hassles in social welfare scheme | For the social welfare scheme under the TB program, patients complained of requirement of quarterly submission of documents which hampered their job timings. |
|  | Insufficient cash assistance | The existing cash assistance under the direct benefit transfer program and social welfare scheme for TB, and the transport reimbursement for HIV was perceived to be insufficient by the patients. |
| Employment interruption | Inability to work | Due to weakness during the initial stages of the diseases, patients are unable to go to work. In certain families, such patients are the sole earners and income of the entire family stops due to the illnesses. |
|  | Wage loss | Patients, generally who are daily wage earners, have to lose their wages for the days of hospital visits and hospitalization whenever required. In addition, due to the debilitating nature of the diseases, they are unable to go to work. |
|  | No provision of sick leave | Workers in the unorganized sector or, daily wage workers do not have the provision of sick leaves. They have to forfeit their wages during hospital visits. |
|  | Loss of working time | The timings of OPD and the working time of the patients generally coincide. This leads to loss of their working time for at least half of the day. |
|  | COVID-related work loss | Due to COVID-19 lockdown, some patients complained of loss of work and loss of wages. |
|  | Job loss (HIV) | For the care of HIV, patients have to visit tertiary care hospital on monthly basis. Patients are unable to avail of leaves from their workplace on regular basis. Some patients also complain of loss of employment due to HIV and the requirement of monthly visits. |

TB: Tuberculosis; HIV: Human Immunodeficiency Virus, PHI: Public Health Institution; OPD: Out-Patient Department, AKT: Anti-Koch’s Treatment, COVID-19: Coronavirus Disease-2019

Supplementary Table 3: Description of solutions for increased costs incurred by patients with TB co-infected with HIV, as perceived by program functionaries and TB-HIV patients

| **Solutions for increased costs of TB-HIV** | | |
| --- | --- | --- |
| **Categories** | **Code** | **Description of code** |
| Improved care | Decentralized care | Patients have to travel to tertiary care hospitals for medicines of HIV or for follow-up visits. If medicines for HIV are available at the nearby government health centers, then patients need not travel long distances and can save on their transport fare as well as can continue their jobs. |
|  | High clinical suspicion | Medical Officers (especially at primary health centers) need to have a high clinical suspicion for tuberculosis. This would help in early case detection and early initiation of treatment. |
|  | Confidentiality of disease status | Staff at government health centers should maintain confidentiality of disease status. This would build trust among the patients towards government health centers and prevent them from seeking care in private sector. |
|  | Simplified reporting | Program functionaries complain of a very lengthy process of reporting due to addition of online data entry portal. This consumes a lot of their time with little time for actual field visits. |
| Improvements in private sector | Mandatory government AKT | Program functionaries perceived that government should make it mandatory for all patients, even from those seeking care in private sector, to avail anti-tuberculosis treatment (AKT) from government health facilities only |
|  | Public-private partnership | The need for strengthening public-private partnership was emphasized. Under the TB program, patients taking treatment in the private sector can avail all the benefits (including cash assistance) as a patient being treated in the government This would also increase notification of TB cases from the private sector. |
| Create awareness | TB champions | TB champions group, comprising of village leaders, health worker and patients previously treated with TB, was being formed in villages. This group would counsel new patients with TB whenever needed, especially when a patient is reported as lost to follow-up. Patients with TB who have completed their full course of treatment act as role models for patients who are reported as lost to follow-up. |
|  | Counselling on adverse drug reactions | Adverse drug reactions was a common reason for treatment interruption among the patients. Program functionaries stressed upon the need for counselling of patients who report adverse drug reactions so that they do not stop their treatment or shift to private sector. |
|  | Awareness generation for private doctors | Awareness generation among private doctors on various benefits like cash assistance of 500 Indian rupees each for notification and informing outcome of a patient with TB, and newer guidelines like CBNAAT testing (cartridge-based nucleic acid amplification testing) for diagnosis is required. |
|  | Increase faith in government | It is important to increase faith of patients on government This faith would prevent them from seeking care in private sector. |
|  | Guidance on services for private patients | Patients seeking care from private sector need to be guided on services which they can avail from the government like cash assistance, testing for HIV, testing for diabetes, and others. |
|  | Counselling | For co-infected patients, program functionaries felt it important to counsel on newly diagnosed HIV among patients with TB. Especially, it was stressed to counsel that the medicines for HIV are given separately and the need to visit a tertiary care hospital for availing the medicines. |
|  | Awareness generation | Awareness generation is required among the patients, family members, and community regarding the signs/ symptoms of TB, importance of completing treatment, for reducing stigma, seeking public healthcare facilities, and availability of medicines in government - for reducing their costs incurred. |
| Cash and other benefits | Insurance scheme | TB program needs to be integrated with the Prime Minister’s Jan Arogya Yojana (PM-JAY), a scheme for vulnerable people to avail care in private hospitals, so that non-affording patients can avail of free treatment for TB in the private sector. |
|  | Increase in cash assistance | Both program functionaries as well as the patients suggested to increase the cash assistance being provided under the TB program. A patient opined that 500 Indian rupees per month would cover only their transport fare during clinic visits. An increase in cash assistance would help them purchase nutritious food. It would also help cover some of the anticipated wage loss during their treatment. |
|  | Reimbursement of costs | Reimbursement of costs incurred during clinic visits for transport, laboratory investigations, and others should be given. This would encourage them for visiting healthcare facilities whenever needed. |
|  | Quick fund transfers | Faster disbursement of cash assistance under the direct benefit transfer scheme would help the patients. |
|  | Cash assistance | Cash transfer program benefitted the patients in many ways. To some, it helped in expenditures at home or, for routine livelihood or, in coping with costs incurred during their care for TB. |
|  | Timely cash assistance | Timely cash assistance would help the patients, especially the sole earners in their families, cope with the sudden stoppage of income due to TB. |
|  | Social welfare scheme | Patients with TB belonging to a reserved category get assistance of 500 Indian rupees for six months, in addition to the assistance under the direct benefit transfer program |
|  | Transport reimbursement | Patients in rural areas have to reach to tehsil-level for availing services of a designated microscopy center or for reaching tertiary care center. If government provides reimbursement of transport fare for the patients along with an accompanying member, patients would not hesitate to go for clinic visits. |
|  | Assistance for buying nutritious food | Nutritious food is important for maintaining immunity during the illness. Government should provide financial assistance for purchasing nutritious food, especially for TB patients co-infected with HIV. |
|  | Nutritious food kit | Program functionaries try their best to find non-governmental organizations or donors who would help them to provide a nutritious food kit comprising of pulses, dates, oil, and protein powder, especially for multi-drug resistant TB patients. The nutritious food kit would help in boosting their immunity. |
|  | Wage loss compensation | A patient with HIV suggested government to compensate for the loss of wages incurred during their monthly visits to tertiary care hospitals for medicine stock replenishment. |
| Health worker support | Frontline worker involvement | Frontline health workers of public health institutions (PHIs) should be involved in home visits of patients with TB. The TB health workers have a very large geographical area to cover, making it difficult for them to carry out home visits of all TB patients. |
|  | Monitor frontline workers | TB health workers should be given authority to supervise and monitor the activities of frontline health workers of public health institutions (PHIs) related to the TB program. |
|  | Address service-related issues of staff | Contractual recruitment of staff involved in TB program demotivates them. Program functionaries, some working for over 20 years in the TB program, demanded for permanent recruitment. |
|  | Supportive care at home | Frontline health workers can provide supportive care at home to patients with TB. Of special mention was counselling and provision of basic supportive drugs for adverse drug reactions to anti-TB medication. Also, frontline health workers can refer patients to higher center as and when required. |
| Improve government health systems | Increase manpower | A dedicated laboratory technician, under the TB program, posted at primary health centers (PHCs), for sputum microscopy would be beneficial. Currently, some PHCs do not have a laboratory technician and one technician is sometimes given additional charge of a PHC with vacancy. |
|  | Increase X-ray facilities | At some community health centers (CHCs), X-ray machines are non-functional. Where present, the X-ray machines are very old, making it difficult for Medical Officers to diagnose tuberculosis or other findings. Thereby, digital X-ray machines were proposed by program functionaries. |
|  | Strengthen tehsil-level facilities | The community health centers (CHCs) at tehsil-level need strengthening. Increasing specialists, running special OPDs for TB, developing facilities for investigation of multi-drug resistant patients like audiogram, electrocardiogram, and others, would help reduce patients’ visits to tertiary care hospitals. |
|  | Public healthcare seeking | Patients would incur lesser costs if they seek care at government health facilities right from the beginning. |
|  | Treatment at nearby health centers | Treatment, especially for HIV, is available at tertiary care hospitals only. Patients with HIV have to travel every month to tertiary care hospitals for their medicines. Their entire day would be saved, thereby reducing their costs, if treatment (medicines) are made available at nearby health centers. |
|  | Dedicated hospitals/ wards | Dedicated OPDs, wards, hospitals with dedicated staff for tuberculosis was suggested by a program functionary. |
|  | Minimize visits | Through the network of TB health visitors and senior treatment supervisors, patients who have any complaints should first contact these workers who would provide initial advice. Only if required, patients would be called for a clinic visit. |
|  | Bi-directional strengthening | The bi-directional TB-HIV collaborative activities need to be strengthened. The program functionaries agreed that the coverage of bi-directional screening was not 100% and needed strengthening. |
|  | TB-HIV integration | There is no direct monitoring of patients with HIV. Patients with HIV visit the tertiary care hospital on monthly basis to replenish their medicine stock. There is no mechanism to monitor patients in between their hospital visits, as in the case of TB. When a patient is co-infected with TB as well as HIV, the treatment for HIV should be as per guidelines of TB. |
|  | Good quality medicines in government | Patients perceived that if the quality of medicines is improved in government, then it is as good as seeking care in private sector. |
|  | Medicine stock of 2 months (HIV) | Patients suggested to provide two-monthly stock of medicines for HIV, instead of the current monthly replenishment. This would reduce their hospital visits and save their costs incurred for such visits. |
| Home-delivered care | Home-delivered services | Home-delivered services are being provided for patients with TB, but not for patients with HIV. There is no dedicated staff under the HIV program who does home visits. There are link workers in HIV program recruited by private organizations and they are limited to the place where these organizations are located. Unlike for TB, link workers do not provide services in villages. If home-delivered services are provided, then the patients’ visits to clinics can be reduced significantly. |
|  | Early diagnosis nearby | The Village Health and Nutrition Days (days of routine immunization and other services provided at village-level) should be explored as an opportunity for early diagnosis of HIV at the village level, as suggested by a program functionary. Dates of such days/ events should be prominently displayed in the village to inform the community. |
| Timely referral | Referral by private doctors | Prompt referral by private doctors to government health facilities, immediately upon diagnosis of TB/ HIV, would help the patients reduce their costs. Instances were given by patients stating that the private practitioners referred them to government health facility saying that the medicines would be costly in private and some of these medicines would be available at government facility only. |
|  | Early diagnosis | Early diagnosis by private practitioners was suggested by a patient. The patient complained that private doctors order too many reports for which patients have to incur costs, and that only after ordering too many reports, the private doctors are able to diagnose their disease. Thereby, it was suggested that early diagnosis would help reduce patient costs. |

TB: Tuberculosis; CBNAAT: Catridge-based Nucleic Acid Amplification Test; HIV: Human Immunodeficiency Virus; PHC: Primary Health Center; AKT: Anti-Koch’s Treatment; OPD: Out-Patient Department; CHC: Community Health Center; PHI: Public Health Institution
